# Case Report: Anti-CD7 CAR-T cell therapy in two patients with relapsed/refractory acute leukemia of ambiguous lineage with aberrant CD7 expression

**DOI:** 10.3389/fimmu.2026.1961900

**Published:** 2026-10-29

**Authors:** Shuai Zhang, Yuehui Lin

**Affiliations:** Ward 9, Department of Hematology, Beijing Boren Hospital, Beijing, China

**Keywords:** aberrant CD7 expression, acute undifferentiated leukemia, CD7 CAR-T cell therapy, hematopoietic stem cell transplantation, leukemia of ambiguous lineage, mixed phenotype acute leukemia

## Abstract

**Background:**

Acute leukemia of ambiguous lineage (ALAL) presents a formidable therapeutic challenge, marked by high relapse rates and dismal long-term survival. The optimal treatment strategy, particularly for acute undifferentiated leukemia (AUL), remains elusive. CD7-targeted chimeric antigen receptor T-cell (CAR-T) therapy has shown considerable promise in treating T-cell malignancies. Consequently, for patients with relapsed or refractory ALAL who exhibit abnormal CD7 expression, CD7-CAR-T cell therapy represents a promising but still preliminary therapeutic avenue that warrants further investigation. This study aims to evaluate the efficacy and safety of CD7 CAR-T cell therapy in this specific patient population.

**Methods:**

Two patients with relapsed/refractory (R/R) ALAL and aberrant CD7 expression participated in a clinical trial of CD7 CAR-T therapy (YK-CD7BB-BL4–002 vector). Patient 1, diagnosed with mixed phenotype acute leukemia (MPAL, B/My), received autologous CD7 CAR-T cells (9.0×10^5cells/kg) after lymphodepleting chemotherapy. Patient 2, diagnosed with acute undifferentiated leukemia (AUL), was treated with donor-derived CD7 CAR-T cells (10.4×10^5cells/kg). Safety and efficacy were evaluated through bone marrow assessment, flow cytometry, and molecular monitoring.

**Results:**

Both patients attained swift CD7-negative morphological complete remission (CR) with flow-MRD negativity, but low-level detectable *PICA*LM-*MLLT10* fusion transcript (0.14%) by RT-qPCR persisted on day 15 in Patient 2 (above the RT-qPCR LoD of 0.01%); Patient 1 achieved molecular remission (*RPN1-MECOM* undetectable) on day 19. Patient *1* proceeded to allogeneic hematopoietic stem cell transplantation (allo-HSCT)within two weeks of remission but suffered a molecular relapse at 3 months post-transplant, ultimately leading to death. Patient 2 achieved flow-MRD-negative marrow remission with residual low- level *PICALM-MLLT10* transcript; however, peripheral blood *PICALM-MLLT10* fusion gene levels surged dramatically by day 42, indicating extramedullary/systemic molecular relapse despite persistent BM flow-MRD negativity. Both patients developed profound myelosuppression and prolonged cytokine release syndrome (CRS). Patient *2* experienced grade 3 CRS and fatal neurotoxicity most consistent with probable ICANS accompanied by progressive cranial nerve deficits.

**Conclusions:**

CD7-directed CAR-T therapy can induce rapid morphological and flow-MRD-negative remission in these two patients with heavily pretreated R/R ALAL and aberrant CD7 expression. Nevertheless, early relapse patterns and significant toxicities—notably severe neurotoxicity in the transplantation context —highlight the imperative for meticulous patient selection, robust supportive care, and combinatorial strategies to consolidate remission. Large-scale prospective trials are urgently warranted to define the definitive role of this therapy in ALAL.

Clinical trial registration:

## 1 Introduction

Acute leukemia of ambiguous lineage (ALAL) arises from multipotent hematopoietic progenitors, lacking clear unilineage differentiation or definitive lymphoid or myeloid characteristics. ALAL constitutes 2% to 5% of all acute leukemia cases and is notably rarer in pediatric populations (1). It is classified as a high-risk leukemia, marked by a formidable relapse rate and a profoundly poor long-term prognosis. A registry analysis from the SEER database revealed that, compared to patients with acute lymphoblastic leukemia (ALL) or acute myeloid leukemia (AML), patients with MPAL faced a 59% and 26% increased risk of death, respectively (2).A retrospective real-world outcome analysis of MPAL patients in the United States reported a stark median overall survival of only 4.2 months and a daunting 30-day mortality rate as high as 33% (3). Acute undifferentiated leukemia, which fails to express known lineage-specific markers, carries an exceptionally grim prognosis, with a median overall survival of merely 9 months—starkly shorter than that of ALL and AML (4, 5).

In the field of hematologic malignancy treatment, relapsed and refractory diseases have long presented significant clinical challenges. With the rapid development of immunotherapy technologies, CAR-T therapy has brought new hope to such patients. Among these, CD7-targeted CAR-T therapy has gradually become a research and application hotspot due to its specific expression advantages in T-cell malignancies (6), peripheral T-cell lymphoma (PTCL) (7), and certain AML (8, 9).CD7 is a single-chain transmembrane glycoprotein primarily expressed on the surface of immature T cells and natural killer (NK) cells (10, 11), and it is also expressed on the surface of certain leukemia/lymphoma cells (12, 13). It serves as a critical cell-surface marker for hematologic malignancies. CD7 is expressed by tumor cells in over 95% of patients with T-cell acute lymphoblastic leukemia (T-ALL) and T-cell lymphoblastic lymphoma (T-LBL) (14). In the PTCL subtype, the CD7 expression rate ranges from 10% to 50% (e.g., 25%–50% in PTCL-NOS, 25%–50% in AITL, and *10*%–50% in ALCL) (15, 16). Approximately 30% of patients with AML also show CD7-positive expression (17–19).CD7 is expressed in limited quantities in normal mature cells and is exclusively present in immature T cells and NK cells, providing a “tumor-specific” window for targeted therapy. CAR-T therapy targeting CD7 can directly recognize and kill tumor cells expressing CD7, offering a novel therapeutic breakthrough for patients with relapsed or refractory hematologic malignancies. For CD7-positive ALAL, CD7-directed chimeric antigen receptor T-cell (CAR-T) therapy may represent a potentially effective treatment strategy, as previously reported in patients with CD7-positive AML (8, 20). *I*t provides us with the following insight: when different diseases share the same molecular targets or immunological markers, we may adopt relevant immunotherapy targeting these targets, potentially achieving favorable therapeutic outcomes. This article presents reports on the use of CD7 CAR-T regimens in ALAL patients. This innovative approach aims to explore new pathways for potential novel therapeutic strategies.

## 2 Methods

### 2.1 Study approval and clinical protocols

Two patients with ALAL presenting with aberrant CD7 expression were enrolled in the clinical trial of CD7 CAR-T therapy. This trial was approved by the Beijing Boren Hospital. Written informed consent was obtained from all participants. Prior to CAR-T cell infusion, all patients underwent lymphodepleting chemotherapy from day -5 to day -3. Day 0 was defined as the day of CAR-T cell infusion. Eligible patients had histologically confirmed *R*/R ALAL (MPAL or AUL per WHO/ICC criteria), measurable disease, defined as ≥5% bone marrow blasts by morphology, detectable minimal residual disease (MRD) by multiparameter flow cytometry and/or disease-specific RT-qPCR, or measurable extramedullary disease on imaging, and adequate organ function to tolerate lymphodepleting chemotherapy; patients with uncontrolled active infection or poor performance status were excluded. Controlled graft-versus-host disease on stable immunosuppression was not considered an exclusion criterion. Aberrant CD7 positivity was predefined as surface CD7 expression on ≥20% of leukemic blasts by multiparameter flow cytometry. CRS and ICANS were graded according to the ASTCT consensus criteria (21); cytopenias were graded per EHA/EBMT consensus criteria, and all other adverse events per CTCAE v5.0. All study days refer to days relative to CAR- T cell infusion (day 0). A graphical overview of the disease course, prior treatment approach, lymphodepleting conditioning, and post-infusion monitoring of both patients is provided in **Figure 1**.

**Figure 1:**
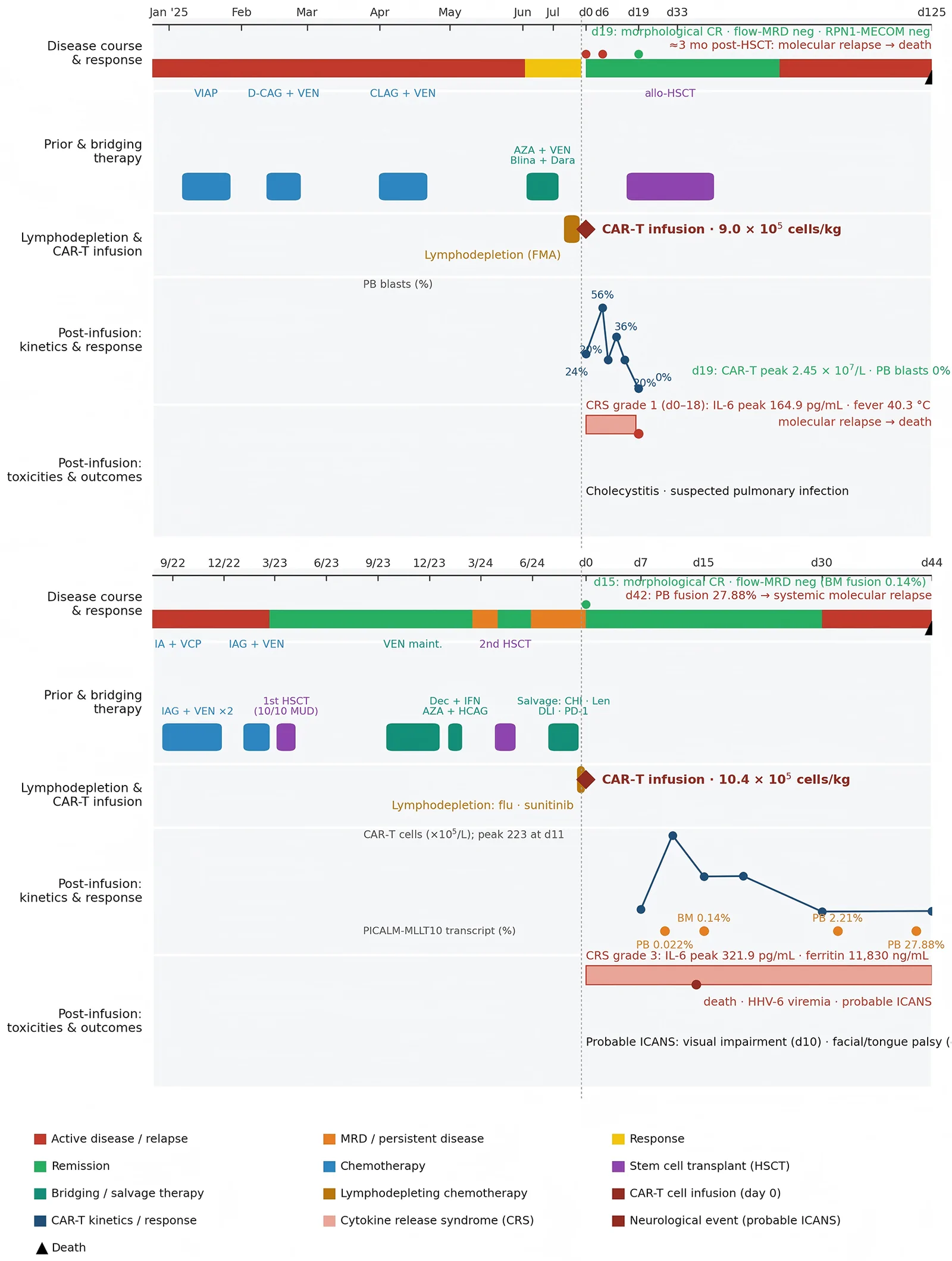
Graphical treatment timeline of the two patients with relapsed/refractory acute leukemia of ambiguous lineage (ALAL) and aberrant CD7 expression who received CD7-targeted chimeric antigen receptor T-cell (CD7 CAR-T) therapy. (**(A)** Upper panel) Patient 1 (31-year-old woman; mixed-phenotype acute leukemia, B/myeloid [MPAL, B/My]), treated with autologous CD7 CAR-T cells (9.0 × 10^5^ cells/kg). (B, Lower panel) Patient 2 (35-year-old woman; acute undifferentiated leukemia [AUL] harboring the PICALM-MLLT10 fusion), treated with donor-derived CD7 CAR-T cells (10.4 × 10^5^ cells/kg). For each patient, the swimlanes depict, from top to bottom: the disease course and response status; prior and bridging therapies; lymphodepleting chemotherapy and CAR-T cell infusion (day 0); and the post-infusion course, including CAR-T cell kinetics, response assessments, toxicities, and outcomes. Time is expressed in calendar months on the left axis and in days relative to CAR-T cell infusion (day 0) on the expanded right axis; the time axis is not drawn to scale. Colored bars in the disease-course lane indicate active disease or relapse (red), minimal residual disease (MRD) or persistent disease (orange), response (yellow), and remission (green). Both patients achieved rapid morphological complete remission (CR) with flow-cytometric MRD negativity by day 19 (Patient 1) and day 15 (Patient 2) after infusion, but both subsequently relapsed: Patient 1 developed molecular relapse approximately 3 months after consolidative allogeneic hematopoietic stem cell transplantation (allo-HSCT) and died; Patient 2 developed systemic molecular relapse by day 42 and died on day 44 with probable immune effector cell-associated neurotoxicity syndrome (ICANS) and HHV-6 viremia. AZA, azacitidine; BM, bone marrow; CHI, chidamide; CR, complete remission; CRS, cytokine release syndrome; Dara, daratumumab; DLI, donor lymphocyte infusion; FMA, fludarabine, mitoxantrone, cytarabine; HSCT, hematopoietic stem cell transplantation; IFN, interferon; MRD, minimal residual disease; MUD, matched unrelated donor; PB, peripheral blood; VEN, venetoclax.

Response definitions: morphological remission (CR) was defined as <5% bone marrow blasts on morphological examination; flow-MRD negativity was defined as the absence of detectable aberrant blasts by multiparameter flow cytometry (corresponding to residual disease below the ~0.1% detection threshold); molecular remission by RT-qPCR required the relevant fusion-gene transcript (*PICALM-MLLT10* or *RPN1-MECOM*) to be undetectable, i.e., below the analytical limit of detection (LoD) of the assay (0.01% for the *PICALM-MLLT10* RT-qPCR assay).

### 2.2 CAR-T cell manufacturing

The preparation of CD7 CAR-T cell products uses donor- or autologous-derived T cells as starting materials, which are genetically modified using the YK-CD7BB-BL4–002 CD7 CAR lentiviral vector. The vector utilizes IntraBlock^®^ technology (patent pending), which incorporates a CD7-binding single-chain variable fragment (scFv) derived from a murine anti-CD7 monoclonal antibody hybridoma, fused to a KDEL endoplasmic reticulum (ER) retention signal sequence. This design promotes intracellular retention of CD7 molecules and suppresses surface CD7 expression on transduced T cells, thereby preventing CAR-T cell fratricide. The CAR construct comprises a CD7-targeting domain, a 4-1BB costimulatory domain, and a CD3ζ signaling domain.

The specific preparation procedure is as follows:

Cell collection and activation: Peripheral blood mononuclear cells (PBMCs) are isolated from peripheral blood via leukocyte separation techniques and subjected to overnight *in vitro* activation stimulation using anti-CD3/anti-CD28 monoclonal antibodies provided by T&L Biotechnology Co., Ltd.

Lentiviral transduction: On the day after activation, the CD7 CAR lentiviral vector is introduced into activated T cells at a multiplicity of infection (MOI) of 5.

Extracellular culture expansion: Transduced cells are seeded in TexMACS serum-free medium produced by Miltenyi Biotec GmbH. Throughout the process, 300 IU/mL interleukin-2 (IL-2) is added to maintain cell proliferation, and the cells are cultured continuously until transfusion.

Key quality control testing:

Cell function and viability verification: Flow cytometry is used to assess transduction efficiency (calculated as the percentage of CAR-positive cells in CD3+ T cells) before infusion, and cell viability is determined by the trypan blue exclusion method.

Microbiological safety screening: All CD7 CAR-T cell products must undergo comprehensive microbiological testing, including endotoxin content determination, bacterial contamination screening, and mycoplasma infection detection, to ensure clinical application safety.

### 2.3 Flow cytometry

Flow cytometry employs fluorescein-labeled monoclonal antibodies and a flow cytometer to detect cell antigen expression, cell size, and the quantity of intracellular granules. This enables the differentiation between normal lymphocytes and CAR-T cells. The monoclonal antibodies and the associated reagents are sourced from BD Company, USA, while the CAR-T antibodies are supplied by the vector company. The instrument utilized is the BD FACSCalibur flow cytometer from the USA, and the analysis is carried out using CellQuest Pro software or FlowJo software.

### 2.4 ELISA

Serum levels of IL-6, IL-8, and IL-10 were detected using commercial ELISA kits (QuantoBio, Registration Number for Medical Devices: TJ-FDA-Approved; Production License Number: TJ-FDA-20200001), and the protocols were followed according to the manufacturer’s instructions.

## 3 Results

### 3.1 Patient characteristics

Patient *1* was a 31-year-old woman who presented with marked leukocytosis (WBC 142.59×10^9/L, 85.3% monocytes), severe anemia (hemoglobin 59 g/L), and profound neutropenia. Bone marrow examination confirmed mixed-phenotype acute leukemia (MPAL, B/My) (**Table 1**).Induction chemotherapy was initiated on January 21, 2025, but the day-15 evaluation showed no response. Despite additional treatment cycles, the disease remained refractory, with only partial remission achieved. By late May 2025, morphological relapse was evident in the bone marrow, and flow cytometry detected abnormal blasts. Disease progression accelerated rapidly: by June 15, bone marrow blasts had surged to 34.44% by flow cytometry, with an immunophenotype expressing CD117, myeloid markers (CD13, CD33), CD56, CD7, and dim/partial B-cell markers (CD19, CD10, CD22), accompanied by 36% circulating blasts in the peripheral blood. Molecular analysis at this time also revealed newly acquired *RP*N1- *MECOM* gene alterations(**Table 1**).Given the high tumor burden and rapid doubling time, cytoreductive therapy with azacitidine plus venetoclax was commenced. Blinatumomab and daratumumab were subsequently added to target the observed CD19 and CD38 expression, resulting in a modest reduction of peripheral blood blasts. On July 3, 2025, lymphodepleting chemotherapy with FMA (fludarabine, mitoxantrone, cytarabine) was administered. Cerebrospinal fluid analysis revealed 5 abnormal blasts among 53 total cells. The patient then received a single infusion of autologous CD7 CAR-T cells at a dose of 9.0×10^5cells/kg in July 2025(**Table 1**).

**Table 1:** Baseline clinical characteristics of patients and cell manufacture.

Baseline clinical characteristics of patients and cell manufacture		
Characteristic	Patient 1	Patient 2
Age	31	35
Sex(gender)	F	F
Morphology(BM)	93%	85%
Immunophenotype(BM)	89.75% of cells expressed CD34, CD117, CD13bri, CD33, HLA- DRbri, CD38, CD7dim (97.6%), CD19dim, and CD79a dim; some cells expressed TdT and CD22dim;	88% of cells expressed CD33, CD7, CD5bri; CD34, CD123, CD38, CD99, and CD45; partial expression of HLA-DR(55.8%) and CD19dim(5.4%);
Karyotype	45,XX,inv(3)(q21q26.2),-7[19]/46, XX[*1*]	45-46,del(5)(q13q21),del(11q21),del(13q21)[6]/46,XX[14]
Gene mutations	*IKZF1*、*WT1*/*ABL* 、*CUX1*	*PICALM-MLLT10* 、*PHF6*、*ASXL2*、*ETV6*
Diagnosis	MPAL(B/My)	AUL
Treatments and response	Multi-line chemotherapy, persistent disease.	Multi-line chemotherapy+HSCT,ultimate molecular relapse and radiographic progression.
Treatments (before CAR-T)	VEN+azacytidine+BiTE+Dara ;persistent disease	CT+VEN+DLI+CHI+PD-1 Inhibitor+Lenalidomide ;Persistent molecular positivity
EMD(before CAR-T)	Mild metabolic space-occupying lesion in theanterior mediastinum (PET-CT)	Increased metabolic activity reveals within the lymph nodes at the thoracic inlet, indicating potential tumor-related lesions (PET-CT).
CNSL(before CAR-T)	YES	NO
Allo-HSCT(before CAR-T)	NO	YES(Two allogeneic HSCTs)
Karyotype(before CAR-T)	46,XX,inv(3)(q21q26)[4]/45,idem,-7[cp16]	46,XX[20]
Gene mutations(before CAR-T)	*RPN1-MECOM*;KRAS;CCND3; *IKZF1*	*PICALM-MLLT10*
Tumour burden(before CAR-T, Immunophenotype, BM)	34.44%	0.38%
Immunophenotypes(positive expression, or dim/partial expression as indicated)	CD34+ 、CD45dim、CD117、CD13、CD33、CD38、HLA-DR、 CD56、CD7 、CD8ldim, CD19、CD10、CD22、CD371, Cytoplasmic CD79a (dim)	CD7 、CD45dim、CD34、CD33、CD38、 CD56、CD70、CD371
CD7 expression levels on leukemia blasts(%)	93.94%	100.0%
T-cell Donor Origin	Autologous	Donor
Cell viability (%)	74.4%	74.5%
CD3+T-cells in lymphocytes (%)	97.2%	85.8%
Transduction efficiency (%)	50.4%	80.0%
Cryopreserved Prior to Infusion	No	No
QualityControl(endotoxin detection,Mycoplasma test andmicroscopy)	Qualified	Qualified
Lymphodepleting Chemotherapy	F*+MA	F*+Sunitinib
Available CAR-T cells(×10^5/kg)	9.0	10.4
Infused cell dose (×10^5/kg)	9.0	10.4

F, female; CT, chemotherapy; HCT, hematopoietic cell transplantation; EMD, extramedullary disease; BM, bone marrow; VEN, venetoclax; DLI, donor lymphocyte infusion; CHI, chidamide; CNSL, central nervous system leukemia; BiTE, blinatumomab; Dara, daratumumab; MA (mitoxantrone, cytarabine); F*, Fludarabine.

Patient 2,a 35-year-old woman presented on August 15, 2022, with epigastric symptoms and was diagnosed with acute undifferentiated leukemia (AUL) harboring a *PICALM-MLLT10* (formerly *PICALM-AF10*) fusion and mutations in *PHF6*, *ASXL2*, and *ETV6*(**Table 1**). After initial chemotherapy yielded only partial response, she underwent a 10/10 matched unrelated donor transplant on March 28, 2023, achieving complete donor chimerism. However, molecular relapse was confirmed by March 2024 despite post-transplant venetoclax and targeted therapy. A second haploidentical transplant from her mother (5/10 matched) was performed on April 11–12, 2024, but molecular relapse recurred by June 13. Significant graft-versus-host disease (GVHD) developed, requiring immunosuppression adjustments. By June 25, the *PICALM-MLLT10* fusion was detectable in both bone marrow and peripheral blood, with 0.14% abnormal blasts (**Table 1**). Over the following months, multiple salvage strategies—including decitabine, venetoclax, lenalidomide, lymphocyte reinfusion, and PD-1 therapy—failed to clear minimal residual disease. By August 21, bone marrow showed 0.5% blasts morphologically and 0.38% by flow cytometry (CD7+ primitive phenotype), with the fusion gene at 0.25%.On September 4, 2024, she received fludarabine and sunitinib (Days 1–3). On September 10, she was reinfused with donor-derived CD7 CAR-T cells at 10.4 × 10^5 cells/kg (**Table 1**).

### 3.2 The kinetics of CD7 CAR-T cell expansion in patients and objective responses

Patient *1*, Based on dynamic monitoring data collected from days *0* to 19 post-infusion, CD7 CAR-T cells in peripheral blood demonstrated classic kinetic characteristics: an initial phase of low presence, followed by rapid expansion, a distinct peak, and subsequent decline. From day 0 to day 6, detectable cell levels were virtually negligible; however, by day 6, the proportion of blast cells in peripheral blood had surged to 56%. Throughout the period from day 6 to day 14, while CAR-T cells underwent exponential expansion, the proportion of peripheral blast cells exhibited significant volatility— decreasing to 20% by day 8, rising to 36% at day 11, falling again to 20% by day 14, and ultimately dropping to 0% by day 19 (**Table 2**; **Figures 2A, C**). On day 19, CAR-T cell expansion culminated at its peak(2.45×10^7/L), an event that coincided with the conversion of bone marrow morphology, minimal residual disease (MRD), and the molecular marker *RPN1-MECOM* to negative status, signaling the patient’s achievement of complete molecular remission (**Table 2**; **Figures 2A, C**). Throughout the entire observation period up to day 19, all efficacy parameters remained stable, with no evidence of tumor recurrence. These findings provide compelling evidence for a direct spatiotemporal link between the peak of *in vivo* CAR-T cell expansion and the onset of early, deep remission.

**Table 2:** The kinetics of CD7 CAR-T cell expansion and objective responses in Patient *1.*.

The kinetics of CD7 CAR-T cell expansion and objective responses in Patient 1					
Time point (day post-infusion)	Blast (PB)	*RPN1-MECOM* (BM)	Immunophenotype (BM)	Morphology (BM)	CD7-CART cell (×10^5/L)
d0	24%	1.29%	34.44%	35.5%	–
d6	56%	–	–	–	–
d7	–	–	–	–	0.501
d8	20%	–	–	–	–
d11	36%	–	–	–	–
d12	–	–	–	–	0.563
d14	20%	–	–	–	5.78
d19	*0*	*0*	*0*	*0*	245

PB, Peripheral blood; BM, Bone marrow.

**Figure 2:**
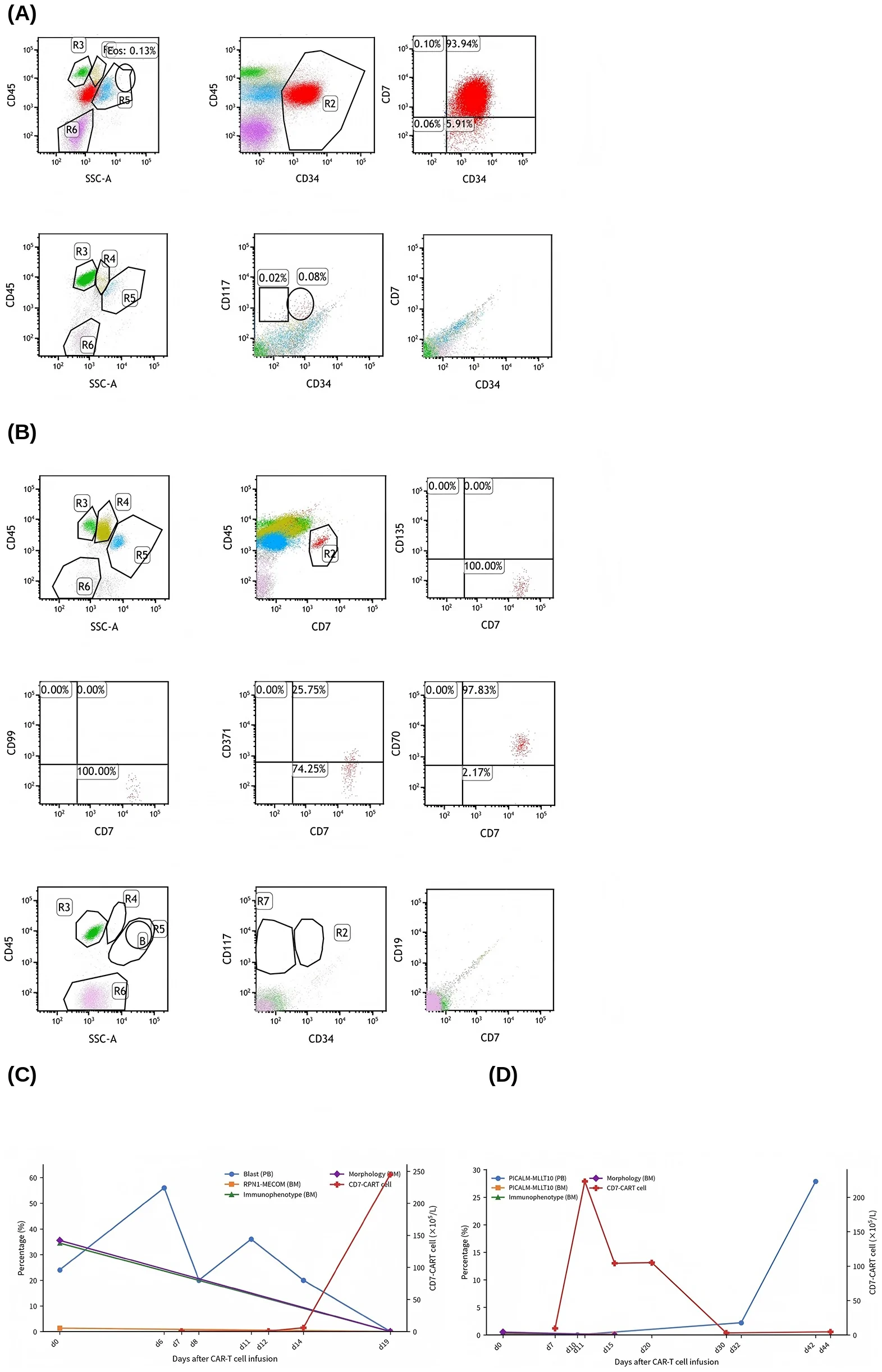
**(A)** Changes in CD7+ leukemia blasts in the bone marrow of Patient 1 following CAR-T cell infusion. Row 1 corresponds to the day of reinfusion (Day 0); Row 2 corresponds to the day of reinfusion (Day 19). **(B)** Changes in CD7+ leukemia blasts in the bone marrow of Patient 2 following CAR-T cell infusion. Row 3 and Row 4 corresponds to the day of reinfusion (Day 0); Row 5 corresponds to the day of reinfusion (Day 15). **(C)** The kinetics of CD7 CAR-T cell expansion and objective responses in Patient 1 (Row 6, Column 1). **(D)**The kinetics of CD7 CAR-T cell expansion and objective responses in Patient 2 (Row 6, Column 2).

Patient 2, Based on dynamic monitoring data from days *0* to 44 post-infusion, the patient’s pre-treatment (day0) assessment revealed a *PICALM-MLLT10* fusion gene positive rate of 0.049% in peripheral blood and 0.25% in bone marrow. The bone marrow minimal residual disease (MRD) level was 0.38%, and morphological examination showed a promyelocyte proportion of 0.5%. Following CAR-T cell infusion, rapid *in vivo* expansion ensued: by day 7, peripheral blood CD7 CAR-T cells reached 9.38 × 10^5/L; they peaked at 223 × 10^5/L on day 11, then gradually declined to 104 ×10^5/L by day 15. Levels remained stable at 105 × 10^5/L on day 20, dropped to 2.82 × 10^5/L at day 30, and further decreased to 4.42 × 10^5/L by day 44. Regarding therapeutic efficacy, by day 15, bone marrow MRD had fallen to 0%, morphological remission was achieved (CR), and bone marrow *PICALM-MLLT10* levels had decreased to 0.14%, indicating residual detectable molecular disease rather than complete molecular remission: although the marrow was flow-MRD negative (below the ~0.1% flow-cytometric detection threshold), the 0.14% transcript level remained above the analytical limit of detection (LoD) of the *PICALM-MLLT10* RT-qPCR assay (0.01%). In contrast, peripheral blood *PICALM-MLLT10* levels initially dropped transiently to 0.022% at day 10, but subsequently rebounded to 2.21% by day 32, before surging dramatically to 27.88% at day 42. This pattern suggested extramedullary/systemic molecular relapse or even progression following CAR-T therapy, despite the bone marrow remaining in a flow-MRD-negative remission state at that time (**Table 3**; **Figures 2B, D**).

**Table 3:** The kinetics of CD7 CAR-T cell expansion and objective responses in Patient 2.

The kinetics of CD7 CAR-T cell expansion and objective responses in Patient 2					
Time point (day post-infusion)	*PICALM-MLLT10*(PB)	*PICALM-MLLT10*(BM)	Immunophenotype(BM)	Morphology(BM)	CD7-CART cell(×10^5/L)
d0	0.049%	0.25%	0.38%	0.5%	–
d7	–	–	–	–	9.38
d10	0.022%	–	–	–	–
d11	–	–	–	–	223
d15	–	0.14%	*0*	*0*	104
d20	–	–	–	–	105
d30	–	–	–	–	2.82
d32	2.21%	–	–	–	–
d42	27.88%	–	–	–	–
d44	–	–	–	–	4.42

PB, Peripheral blood; BM, Bone Marrow.

### 3.3 Adverse effects of CD7 CAR-T cell therapy in patients

Both patients presented with the following core toxicity profile:

The most formidable clinical challenge arose from profound and unrelenting myelosuppression, culminating in refractory pancytopenia. Following transfusion, both patients precipitously developed critically low white blood cell counts (<0.05×10^9/L) and absolute neutropenia, while their platelet counts stubbornly persisted below <10–20×10^9/L for several weeks (**Figures 3A, E**). In Patient 1, hemoglobin levels plunged to a nadir of 37 g/L, compounded by ineffective platelet transfusion (**Figure 3A**). This profound toxicity originates from a confluence of factors: prior damage inflicted by chemotherapy/transplantation, lymphodepleting conditioning regimens, and the potential cytotoxic impact of CD7 CAR-T cells on the hematopoietic microenvironment. Consequently, both patients became dependent on long-term, sustained component blood transfusions for supportive care.

**Figure 3:**
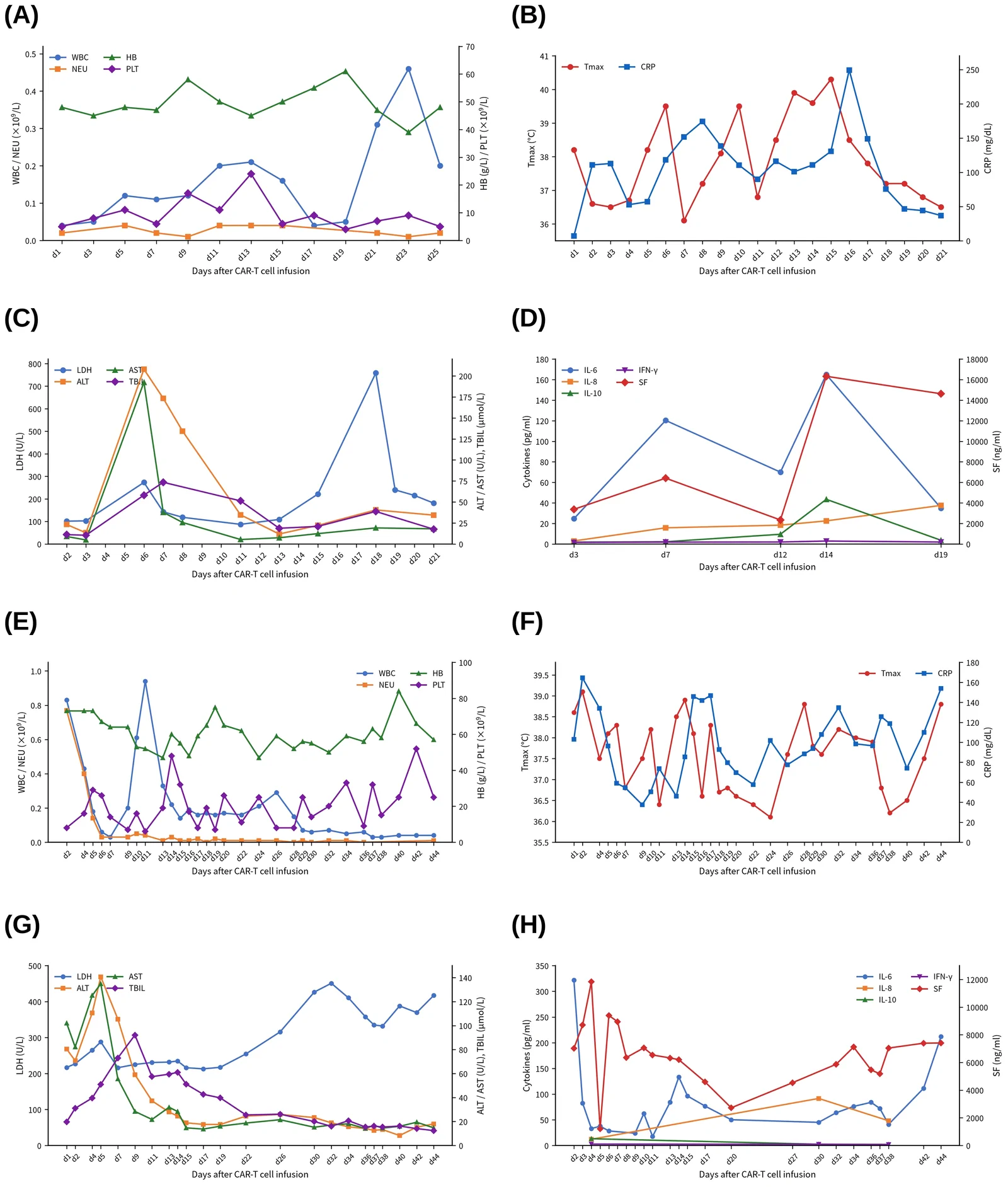
Patient 1: **(A)** Complete blood count trends. **(B)** Temperature and C-reactive protein. **(C)** Liver function tests and lactate dehydrogenase. **(D)** Cytokine and inflammatory markers (including IL-6, IL-8, IL-10, ferritin, and IFN-γ). Patient 2: **(E)** Complete blood count trends. **(F)** Temperature and C-reactive protein. **(G)** Liver function tests, lactate dehydrogenase and creatinine. **(H)** Cytokine and inflammatory markers (including IL-6, IL-8, IL-10, ferritin, and IFN-γ).

A protracted, delayed-onset cytokine release syndrome (CRS): The CRS did not manifest as a single, explosive cytokine storm, but rather as a persistent low-to-moderate inflammatory siege lasting weeks (**Figures 3D, H**). Patient *1* exhibited an IL-6 peak of 164.9 pg/mL (**Figure 3D**; **Table 4**) and endured recurrent fevers until day 18, (with temperatures reaching a maximum of 40.3 °C (**Figure 3B**). Patient 2 developed a high fever (40 °C) from day 2 (**Figure 3F**), with IL-6 soaring to 321.90 pg/mL and ferritin peaking at 11,830 ng/mL (**Figure 3H**; **Table 4**). Both patients required prolonged maintenance therapy with methylprednisolone (4–20 mg/day); any attempt to taper the steroid dosage invariably triggered fever recurrence, underscoring the inflammation’s recalcitrant nature.

**Table 4:** Toxicities of CD7 CAR-T cell therapy.

Toxicities of CD7 CAR-T cell therapy		
Toxicity	Patient 1	Patient 2
CRS grade	*1*	3
Maximal IL-6 (pg/ml)	164.9	321.9
The peak levels of CAR-T cells	2.45×10^7/L	2.23×10^7/L
ICANS	NO	YES
Viremia	NO	HHV-6
Prolonged activated partial thromboplastin time	*2*	*2*
Prolonged prothrombin time	*1*	*2*
Fibrinogen levels are decreased	*2*	3
AST increased	*1*	*2*
ALT increased	*2*	*1*
Elevated bilirubin	1-2	*2*
Creatinine clearance decreased	NO	*2*
Immune effector cell associated hemotoxic effect	3-4	3-4
Pericardial/pleural effusion	NO	YES
Organ Infection	Cholecystitis,pulmonary infection(suspicious)	Parotitis

CRS, Cytokine Release Syndrome; ICANS, Immune effector cell associated neurotoxicity syndrome; Neutropenia and thrombocytopenia were graded using the European Hematology Association (EHA)/the European Society for Blood and Marrow Transplantation (EBMT) consensus criteria. Other adverse events were captured using the Common Terminology Criteria for Adverse Events (CTCAE, version 5.0).

Severe, polymicrobial infections: With their immune defenses critically compromised, each patient battled distinct and refractory infections. Patient 1 primarily suffered organ-specific infections, most notably cholecystitis accompanied by hepatobiliary enlargement and pain, alongside suspected concurrent pulmonary infection and gynecological cyst lesions; anti-infective therapy eventually prompted a gradual improvement in later stages (**Table 4**). Patient 2 faced a more critical course dominated by viral infection combined with mixed pathogens, diagnosed with HHV-6 viremia and mumps, with potential concurrent mixed infections. This led to persistent fever, glandular swelling, and pain that defied anti-infective treatment (**Table 4**). Hepatic and renal dysfunction: Both patients developed hepatic injury during the post-infusion course. Patient 1 had grade 2 ALT and grade 1 AST elevation with grade 1–2 hyperbilirubinemia in the setting of cholecystitis, which gradually resolved with anti-infective and supportive therapy. Patient 2 developed grade 2 AST and grade 1 ALT elevation with grade 2 hyperbilirubinemia during grade 3 cytokine release syndrome. Renal involvement was limited to Patient 2 (grade 2 decreased creatinine clearance), which was managed conservatively; renal function remained normal in Patient 1 (**Figures 3C, G**). Patient 2 also developed pericardial and pleural effusions (**Table 4**).

Critically, Patient *2* developed progressive multiple cranial nerve deficits: visual impairment in the left eye by day 10, right- sided facial paralysis by day 13, bilateral facial paralysis and tongue palsy by day 14, culminating in unequal pupil size (5 mm vs. *2* mm), convulsions, and brain herniation by day 38. These deficits emerged within the typical temporal window of CAR-T expansion (peak on day 11) and grade 3 CRS, meeting ASTCT consensus criteria for probable ICANS. However, because cranial MRI and lumbar puncture were declined by the patient and her family due to rapid clinical deterioration, cerebrospinal fluid findings (cell count, protein, HHV-6 PCR, and cytology) and neuroimaging evidence could not be obtained. Therefore, although HHV-6 viremia was present, this devastating course is best attributed to probable ICANS with possible superimposed HHV-6 encephalitis, CNS leukemia, or viral meningoencephalitis, and the relative contribution of each etiology could not be definitively distinguished (**Table 4**). Formal serial ICE (Immune Effector Cell-Associated Encephalopathy) scoring was not prospectively documented in this patient. Patient 1, by comparison, experienced only headache and dizziness without focal neurological signs. Ultimately, these cumulative and severe toxic reactions drove irreversible disease progression, necessitating treatment termination (**Table 4**).

## 4 Discussion

Acute leukemia of ambiguous lineage (ALAL), encompassing acute undifferentiated leukemia (AUL) and mixed phenotype acute leukemia (MPAL), represents a therapeutically challenging entity characterized by high relapse rates and dismal long- term survival. The absence of consensus treatment protocols, coupled with the poor efficacy of conventional chemotherapy in relapsed or refractory (R/R) settings, underscores an urgent unmet clinical need. In this report, we present the first clinical evidence of CD7-directed chimeric antigen receptor T-cell (CAR-T) therapy in two patients with R/R ALAL exhibiting aberrant CD7 expression. Both patients achieved rapid morphological CR with flow-MRD negativity within 15 to 19 days post-infusion, demonstrating potent on-target antileukemic activity; however, Patient 2 retained a low-level detectable *PICALM-MLLT10* transcript (0.14%) and therefore did not achieve complete molecular remission. However, the durability of remission was limited in both cases, highlighting the critical challenges of antigen escape and disease recurrence that warrant further investigation.

The kinetics of CAR-T cell expansion observed in our patients exhibited classic patterns of initial lag, exponential proliferation, peak expansion, and subsequent contraction. In Patient 1, the CAR-T cell peak coincided precisely with the achievement of molecular remission on day 19, whereas Patient 2 demonstrated a robust expansion peaking at 223 ×10^5/L on day 11, with MRD negativity in bone marrow documented by day 15. This temporal association between peak CAR-T cell activity and disease clearance aligns with mechanistic models of CAR-T-mediated cytotoxicity, wherein sufficient effector-to-target ratios are required for sustained tumor elimination. However, the divergence in clinical outcomes between the two patients warrants careful analysis. Patient 1, who received autologous CAR-T cells at a lower dose (9.0 × 10^5 cells/kg), proceeded to allogeneic hematopoietic stem cell transplantation (allo-HSCT) within two weeks of remission but experienced molecular relapse at three months post-transplant. In contrast, Patient 2, who received donor-derived CAR-T cells at a higher dose (10.4 ×10^5 cells/kg), demonstrated initial flow-MRD-negative marrow remission with residual low-level *PICALM-MLLT10* transcript but subsequently exhibited a dramatic rebound of peripheral blood *PICALM-MLLT10* fusion gene levels, suggestive of extramedullary or systemic molecular relapse. These disparate patterns may reflect differences in disease biology, prior treatment exposure, and the immunological milieu; Patient *2* had undergone two prior allo-HSCT procedures and exhibited refractory graft-versus-host disease (GVHD), which may have compromised both CAR-T cell persistence and endogenous immune surveillance. Donor-derived rather than autologous CAR-T cells were chosen for Patient *2*,because, after two prior allogeneic transplants with persistent marrow failure, adequate autologous T-cell collection was not feasible and a matched donor was already available. Whether donor- versus autologous starting material differentially affects CAR-T expansion, persistence, CRS/ICANS propensity, or disease control cannot be assessed from these two cases, and this comparison remains an important question for future studies.

The limited durability of remission in both patients highlights a central challenge in CD7-targeted immunotherapy: the propensity for disease recurrence. In the context of CD7 CAR-T therapy for AML, Lu et al. reported that among patients achieving CR, those who did not receive consolidative allo-HSCT experienced relapse at a median of 47 to 115 days, with CD7 antigen loss documented in all relapsed cases (8). Similarly, in T-cell acute lymphoblastic leukemia (T-ALL), CD7- negative relapse has been identified as a dominant resistance mechanism following CD7 CAR-T therapy. In our Patient 1, the rapid molecular relapse post-transplantation suggests the presence of residual CD7-negative leukemic stem cells or the emergence of antigen-loss variants under therapeutic pressure. For Patient 2, the discordance between bone marrow MRD negativity and peripheral blood molecular progression raises the possibility of extramedullary disease sanctuary or lineage plasticity mediated by the *PICALM-MLLT10* fusion, which has been associated with aggressive disease behavior. These observations collectively emphasize that CD7 CAR-T therapy, while highly effective at inducing initial responses, may function optimally as a bridging strategy to consolidative allo-HSCT rather than as standalone curative therapy. *I*ndeed, accumulating evidence in T-ALL/LBL suggests that consolidation with allo-HSCT following CD7 CAR-T-induced remission significantly improves progression-free survival compared to CAR-T therapy alone (22). However, these observations are observational associations rather than mechanistic conclusions; multiple confounders, including prior allo-HSCT, conditioning/lymphodepleting therapy, concomitant immunosuppression, infection, and disease burden, could not be controlled. Moreover, paired diagnostic and relapse samples were not available for comparison and longitudinal CAR-T phenotype/persistence was not profiled, so the precise mechanism of relapse—antigen (CD7) escape, inadequate CAR-T persistence, extramedullary sanctuary, or pre-existing CD7-negative clones—could not be definitively distinguished.

The safety profile observed in our cohort reflects the multifaceted toxicities inherent to CD7-directed cellular therapy. Both patients developed severe myelosuppression, necessitating prolonged transfusion support and broad-spectrum antimicrobial therapy. Patient *1* experienced recurrent febrile episodes, cholecystitis, and suspected pulmonary infection during the cytokine release syndrome (CRS) phase, consistent with the profound cytopenias and infectious complications frequently reported following CAR-T therapy in heavily pretreated populations. Patient *2* developed grade 3 CRS characterized by high-grade fever, hyperferritinemia, and transaminitis, which responded to corticosteroid intervention. Of particular concern was the emergence of probable immune effector cell-associated neurotoxicity syndrome (ICANS) in Patient 2, manifesting as bilateral facial paralysis, cranial nerve palsies, and ultimately clinical signs suggestive of impending brain herniation. While ICANS has been reported in approximately 26.9% of patients receiving CAR-T therapy across various hematologic malignancies, the incidence of severe neurotoxicity in CD7 CAR-T trials has generally been low, particularly with naturally selected or fratricide-resistant products (23). Because cranial MRI and lumbar puncture were declined by the patient and her family during rapid clinical deterioration, the diagnosis could not be confirmed by neuroimaging or cerebrospinal fluid analysis and must therefore be regarded as probable rather than definitive. The severe neurological deterioration in Patient 2 may be attributable to multiple converging factors, including donor-derived CAR-T cell-mediated neuroinflammation, reactivation of human herpesvirus-6 (HHV-6) viremia, and the patient’s complex prior history of dual transplantation and GVHD. The presence of subcutaneous nodules and parotid enlargement further complicates the differential diagnosis, raising the possibility of leukemic infiltration, disseminated infection, or immune-mediated tissue injury. These findings underscore the necessity of rigorous neurological monitoring, early neuroimaging, and aggressive multidisciplinary management in patients receiving CD7 CAR-T therapy, particularly those with prior transplant history or evidence of active infection.

Several limitations of this study must be acknowledged. First, the report is based on a two-patient case series from a single institution, precluding generalizable conclusions regarding efficacy and safety. The two patients were also clinically heterogeneous (Patient 1: MPAL with autologous CD7 CAR-T and no prior transplant; Patient 2: AUL with *PICALM-MLLT10* and donor-derived CD7 CAR-T after two prior allogeneic transplants), so they cannot be pooled to estimate response rates or toxicity incidence, and all efficacy and safety observations should be regarded as descriptive and hypothesis-generating rather than definitive. Second, the follow-up duration was insufficient to fully characterize long-term survival outcomes or late toxicities such as secondary malignancies or prolonged B-cell or T-cell aplasia. Third, comprehensive mechanistic studies, including single-cell sequencing of relapsed leukemic clones and longitudinal CAR-T cell phenotyping, were not performed, limiting our ability to definitively identify resistance mechanisms. Fourth, the optimal timing of consolidative allo-HSCT following CD7 CAR-T-induced remission remains undefined; Patient *1* underwent transplantation shortly after achieving remission, yet still experienced early relapse, suggesting that the depth and stability of pre-transplant remission, rather than its mere achievement, may be the critical determinant of transplant success. In addition, longitudinal characterization of the CAR-T products—including CD4/CD8 composition and exhaustion/functional markers such as PD-1, TIM-3, LAG-3 and cytokine polyfunctionality—was not performed, limiting assessment of how product composition and immune phenotype related to *in vivo* kinetics. Finally, follow-up was too short to evaluate long-term efficacy, behavior in chronic progressive disease, or late toxicities, and durable outcomes cannot be inferred from these two patients. Representative flow-cytometric histograms could not be provided because raw FCS files from the screening time points were no longer retrievable from the archive.

Looking forward, several strategies may enhance the therapeutic index of CD7 CAR-T in ALAL. The integration of hypomethylating agents, such as azacitidine or decitabine, may potentiate CAR-T efficacy by upregulating tumor antigen expression and modulating the immunosuppressive microenvironment. A recent case report demonstrated that decitabine priming followed by CD7 CAR-T infusion achieved sustained MRD-negative remission in an MPAL patient relapsed after allo-HSCT (24). Additionally, the development of dual-targeting CAR-T constructs simultaneously engaging CD7 and alternative lineage antigens (e.g., CD19, CD33, or CD38) may mitigate the risk of single-antigen escape. For patients with prior transplant history, the use of universal or donor-derived CD7 CAR-T products offers logistical advantages, though careful titration of immunosuppression and vigilant surveillance for GVHD exacerbation are imperative. Finally, the incorporation of 4-1BB costimulatory domains, as employed in the present study, may promote favorable CAR-T cell persistence and memory formation, though further optimization of the CAR architecture and manufacturing process is warranted.

## 5 Conclusion

In conclusion, this report provides preliminary clinical evidence that CD7-directed CAR-T cell therapy can induce rapid morphological and flow-MRD-negative remission in patients with heavily pretreated, R/R ALAL harboring aberrant CD7 expression. The therapy demonstrates potent on-target antileukemic activity; however, the observed early relapses and significant toxicities, particularly severe neurotoxicity in the context of prior transplantation, highlight the need for cautious patient selection, robust supportive care, and the development of combinatorial or sequential treatment strategies to consolidate and prolong remission. Larger prospective clinical trials with extended follow-up are essential to definitively establish the role of CD7 CAR-T therapy within the therapeutic algorithm for this rare and aggressive leukemia subtype.

## Data Availability

The raw data supporting the conclusions of this article will be made available by the authors, without undue reservation.
